# ECGomics: An Open Platform for AI-ECG Digital Biomarker Discovery

**DOI:** 10.34133/hds.0427

**Published:** 2026-05-04

**Authors:** Deyun Zhang, Jun Li, Shijia Geng, Yue Wang, Shijie Chen, Sumei Fan, Qinghao Zhao, Shenda Hong

**Affiliations:** ^1^ HeartVoice Medical Technology, Hefei 230088, China.; ^2^ National Institute of Health Data Science, Peking University, Beijing, China.; ^3^ College of Integrative Chinese and Western Medicine, Anhui University of Chinese Medicine, Hefei 230012, China.; ^4^ Department of Cardiology, Peking University People’s Hospital, Beijing 100044, China.; ^5^ State Key Laboratory of Vascular Homeostasis and Remodeling, NHC Key Laboratory of Cardiovascular Molecular Biology and Regulatory Peptides, Peking University, Beijing, China.; ^6^ Institute of Medical Technology, Peking University, Beijing, China.; ^7^ Institute for Artificial Intelligence, Peking University, Beijing, China.

## Abstract

**Background:** Conventional electrocardiography (ECG) analysis faces a persistent dichotomy: Expert-defined features provide interpretability but are limited in capturing latent high-dimensional patterns, whereas deep learning approaches achieve strong predictive performance but often lack interpretability and require large annotated datasets. A systematic framework that integrates these paradigms remains needed. **Methods:** We propose ECGomics, a structured and deployable analytical paradigm that deconstructs cardiac electrical signals into 4 interconnected dimensions: Structural, Intensity, Functional, and Comparative. This taxonomy integrates expert-defined morphological metrics with artificial intelligence-derived latent embeddings to generate multidimensional digital biomarkers. **Results:** We operationalized this framework into a scalable ecosystem consisting of a web-based platform, a mobile solution (https://github.com/PKUDigitalHealth/ECGomics), and application programming interface invocation. Across multiple representative clinical scenarios—including atrial fibrillation detection, recurrence prediction after cryoablation, screening of severe coronary stenosis in apparently normal ECGs, and maternal cardiac monitoring—ECGomics demonstrated robust predictive performance while maintaining interpretability and relatively low data requirements. These results validate the flexibility and effectiveness of the proposed multidimensional framework. **Conclusion:** ECGomics establishes an omics-level representation system for ECG analysis, bridging conventional feature engineering and deep learning within a unified taxonomy. By providing a deployable digital biomarker ecosystem, this framework advances scalable precision cardiovascular assessment and data-driven health management.

## Introduction

Electrocardiography (ECG), as a noninvasive, low-cost, and readily accessible physiological signal, has long been regarded as one of the gold standards for cardiovascular disease screening and diagnosis [1,2]. By capturing patterns of cardiac electrical activity, ECG effectively reveals pathological manifestations such as arrhythmias and myocardial ischemia [3]. However, conventional ECG analysis relies heavily on cardiologists’ visual inspection and experience-based judgment (expert rules). This paradigm, centered on explicit features (e.g., ST-segment depression and T-wave inversion), is inherently limited in its ability to capture subtle fluctuations and high-dimensional nonlinear information embedded in ECG signals, leaving a vast amount of latent data value largely unexplored.

With the advent of the big data era in biomedicine, the emergence of omics research paradigms—such as genomics, transcriptomics, and proteomics—has driven transformative advances [4,5]. Rather than focusing on single indicators, these approaches adopt a systems biology perspective to interrogate complex biological systems across multiple dimensions and scales, uncovering hidden patterns and mechanisms [6–8]. This evolution raises a fundamental question: Can ECG signals be conceptualized as a high-dimensional biological data stream and reexamined through an omics-oriented lens to unlock their full life-cycle information content?

In recent years, rapid advances in artificial intelligence (AI), particularly deep learning (DL), have validated the feasibility of this perspective [9–12]. Beyond surpassing human experts in recognizing conventional cardiac pathologies, AI has achieved breakthroughs in identifying generalized ECG features [13,14]. Accumulating evidence demonstrates that deep neural networks can accurately infer biological attributes such as age, sex, and body mass index from ECG signals [15–17] and can effectively screen for noncardiovascular conditions, including hyperkalemia, anemia, diabetes, and renal dysfunction [18–20]. These findings suggest that the heart, as the central driver of systemic circulation and a key target of neurohumoral regulation, produces electrophysiological signals that constitute a holographic projection of whole-body health [21–23]. Such latent features are intrinsically linked to an individual’s genetic background, metabolic state, and phenotypic characteristics, paralleling the way genomics deciphers the fundamental codes of life.

In classical omics disciplines, biological systems are characterized through the systematic transformation of raw signals into structured, high-dimensional representation matrices that enable scalable profiling, cross-sample comparison, and integrative association analysis. ECGomics, we proposed in this study, is proposed within this methodological paradigm. Rather than serving as a metaphorical extension of genomics, it conceptualizes electrocardiographic signals as high-dimensional physiological data streams and formalizes their transformation into standardized, multidimensional digital biomarker vectors organized under a unified and extensible taxonomy. Under this framework, ECGomics represents an interdisciplinary analytical system integrating signal processing, DL, and systems-level biomedical modeling. Its objective is to enable high-throughput, automated, and multidimensional profiling of ECG-derived representations, thereby facilitating systematic investigation of their associations with cardiovascular diseases, systemic conditions, genetic backgrounds, and clinical phenotypes. By elevating ECG from a visually interpreted diagnostic waveform to a structured biological data representation system, ECGomics supports hierarchical organization, interoperability across analytical tasks, and population-level comparative mapping. This transition from parameter extraction to formalized representation establishes a coherent theoretical foundation for advancing data-driven cardiovascular research and precision medicine [24].

## Methods

**Fig. 1.**
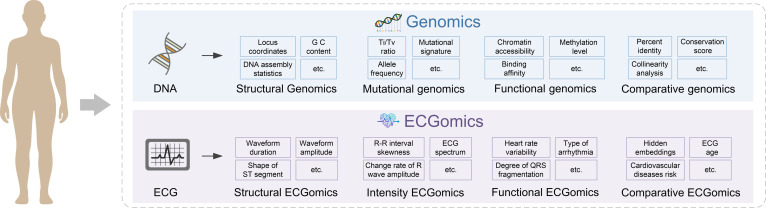
Taxonomic parallel between Genomics and ECGomics. Genomics organizes DNA information into Structural, Mutational, Functional, and Comparative domains. Correspondingly, ECGomics deconstructs cardiac signals into Structural, Intensity, Functional, and Comparative dimensions, enabling transformation of raw electrocardiography (ECG) signals into multidimensional digital biomarkers.

**Fig. 2.**
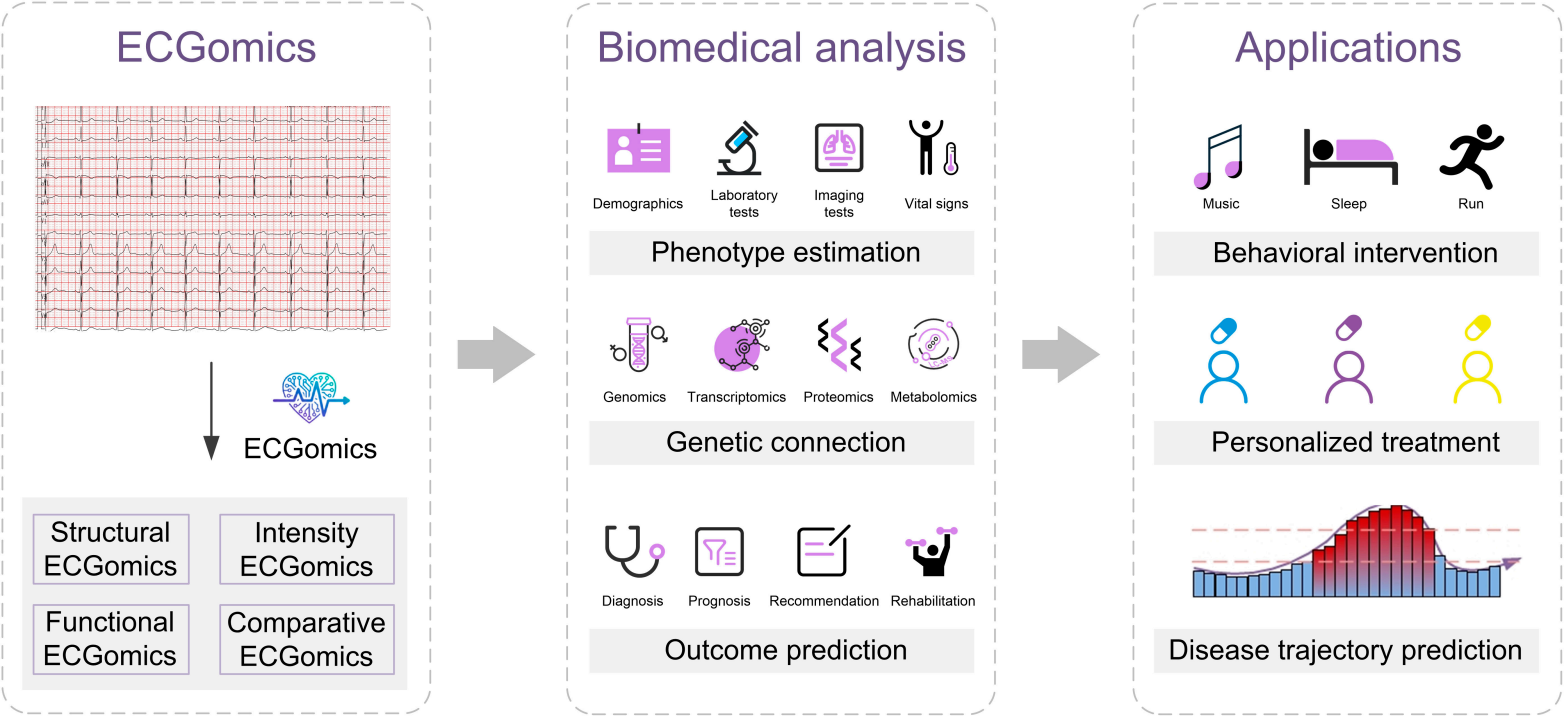
ECGomics-driven workflow from digital biomarkers to clinical translation. The framework comprises 3 layers: (Left) ECGomics data layer for high-throughput extraction of structural, intensity, functional, and comparative ECGomics from raw electrocardiography (ECG) signals; (middle) biomedical analysis layer integrating ECG signatures with clinical, imaging, and multiomics data for deep phenotyping and multiomics association studies; (right) application layer translating insights into precision intervention, personalized therapy, and disease trajectory management.

The concept of ECGomics, as proposed in this study, represents a paradigm shift in cardiovascular medicine, elevating the ECG from a traditional diagnostic tool to a high-throughput, multidimensional digital biomarker. This framework is built upon a taxonomic structure that parallels genomics, aiming to transform raw cardiac electrical signals into structured data representations that capture complex temporal and morphological patterns (Fig. 1). By systematically integrating signal processing with advanced DL foundation models, ECGomics establishes a standardized analytical pipeline that bridges the gap between raw physiological data and actionable clinical insights (Fig. 2).

### ECGomics definition: Four dimensions of ECGomics

The omics-level representation in ECGomics refers to the systematic transformation of raw ECG waveforms into standardized, multidimensional digital feature vectors organized under a unified and extensible taxonomy. Unlike task-specific feature extraction, which isolates predefined parameters for individual predictive objectives, this representation framework is designed to be scalable, modular, and population-aware, enabling consistent application across analytical tasks and study cohorts. Operationally, ECGomics implements this principle by deconstructing cardiac electrical activity into 4 distinct yet interconnected dimensions—Structural, Intensity, Functional, and Comparative ECGomics (Fig. 1). This hierarchical taxonomy integrates expert-defined electrophysiological descriptors with data-driven latent representations, thereby bridging interpretable domain knowledge and deep representation learning within a coherent analytical system.

Structural ECGomics: Structural ECGomics captures electrophysiological features reflecting cardiac structural substrates and conduction pathways, such as P-wave and QRS complex durations and amplitudes. These parameters are derived from expert-defined rules and correspond to the temporal and spatial organization of cardiac depolarization and repolarization, thereby serving as the foundational layer for representing the anatomical and conduction-related architecture of the heart. Analogous to structural genomics—which focuses on elucidating the physical organization of the genome, including chromosomal architecture and gene localization—Structural ECGomics characterizes the electrophysiological architecture of the cardiac cycle, mapping the structural organization of electrical conduction across time and waveform morphology.

Intensity ECGomics: Intensity ECGomics characterizes the intrinsic signal strength, energy distribution, and spectral properties of ECG waveforms, including descriptors such as R-wave amplitude variability, R–R interval skewness, and ECG frequency-domain spectra. These metrics quantify the magnitude, dispersion, and nonlinear complexity of electrical activity. Conceptually, this dimension parallels mutation genomics, which investigates genomic variations in terms of mutation type, frequency, and distribution. While mutation genomics examines variability at the sequence level, Intensity ECGomics quantifies variability and dispersion in electrophysiological signal amplitude and spectral energy, thereby capturing intensity-related heterogeneity within cardiac electrical activity.

Functional ECGomics: Functional ECGomics represents dynamic physiological performance and regulatory processes of the cardiovascular system, incorporating features such as heart rate variability, arrhythmia phenotypes, and QRS fragmentation indices. These indicators assess autonomic modulation, rhythm stability, and conduction integrity, reflecting functional adaptations or dysfunction beyond static structural properties. This dimension is analogous to functional genomics, which investigates gene expression, regulatory networks, and their influence on biological processes. Similarly, Functional ECGomics focuses not merely on structural intervals but on how cardiac electrophysiology dynamically responds to regulatory and systemic influences, thereby revealing the functional state of the cardiovascular system.

Comparative ECGomics: Comparative ECGomics extends analysis to the population level by leveraging AI-derived latent embeddings and large-scale cohort-based reference models. Instead of relying solely on predefined parameters, this dimension quantifies implicit representations, predicted heart age, and disease risk probabilities by benchmarking individual ECG signals against learned population paradigms. This approach parallels comparative genomics, which reveals evolutionary relationships and conserved genomic patterns through cross-species comparison. Analogously, Comparative ECGomics performs cross-individual and population-level benchmarking, identifying shared patterns, latent representations, and probabilistic deviations that may indicate biological aging, disease risk, or phenotypic variation.

The conceptualization of ECGomics is rooted in our extensive prior research and a robust ecosystem of specialized computational tools (Table 1). Specifically, we have developed several state-of-the-art deep neural networks based on the Net1D architecture [25], which were pretrained on diverse, large-scale datasets to ensure high-dimensional feature fidelity. This foundational work directly fuels the 4 dimensions of the ECGomics framework: Structural, Intensity, and Functional ECGomics are primarily derived using the ENCASE [26] and FeatureDB [27] pipelines, which enable the high-throughput extraction of clinically interpretable morphological and physiological features. In contrast, Comparative ECGomics represents a higher-order synthesis, integrating the predictive power of CardioLearn [28] and the vast representation space of ECGFounder [3]. Together, these prior milestones—ranging from expert-driven feature engineering to self-supervised foundation models—provide the multilayered digital biomarker framework required to systematically characterize and quantify the complex relationships between the ECG and the broader systemic health landscape.

**Table 1.** Methodological components organized by ECGomics taxonomy

ECGomics type	Core component	Model type
Structural	ENCASE	Expert-defined framework
	FeatureDB	Signal processing module
Intensity	FeatureDB	Signal processing module
Functional	FeatureDB	Signal processing module
	CardioLearn	Supervised deep model
	ECGFounder	Supervised deep model
Comparative	CardioLearn	Supervised deep model
	ECGFounder	Supervised deep model

### AI-ECG digital biomarker: Perspective of digital biomarkers

**Fig. 3.**
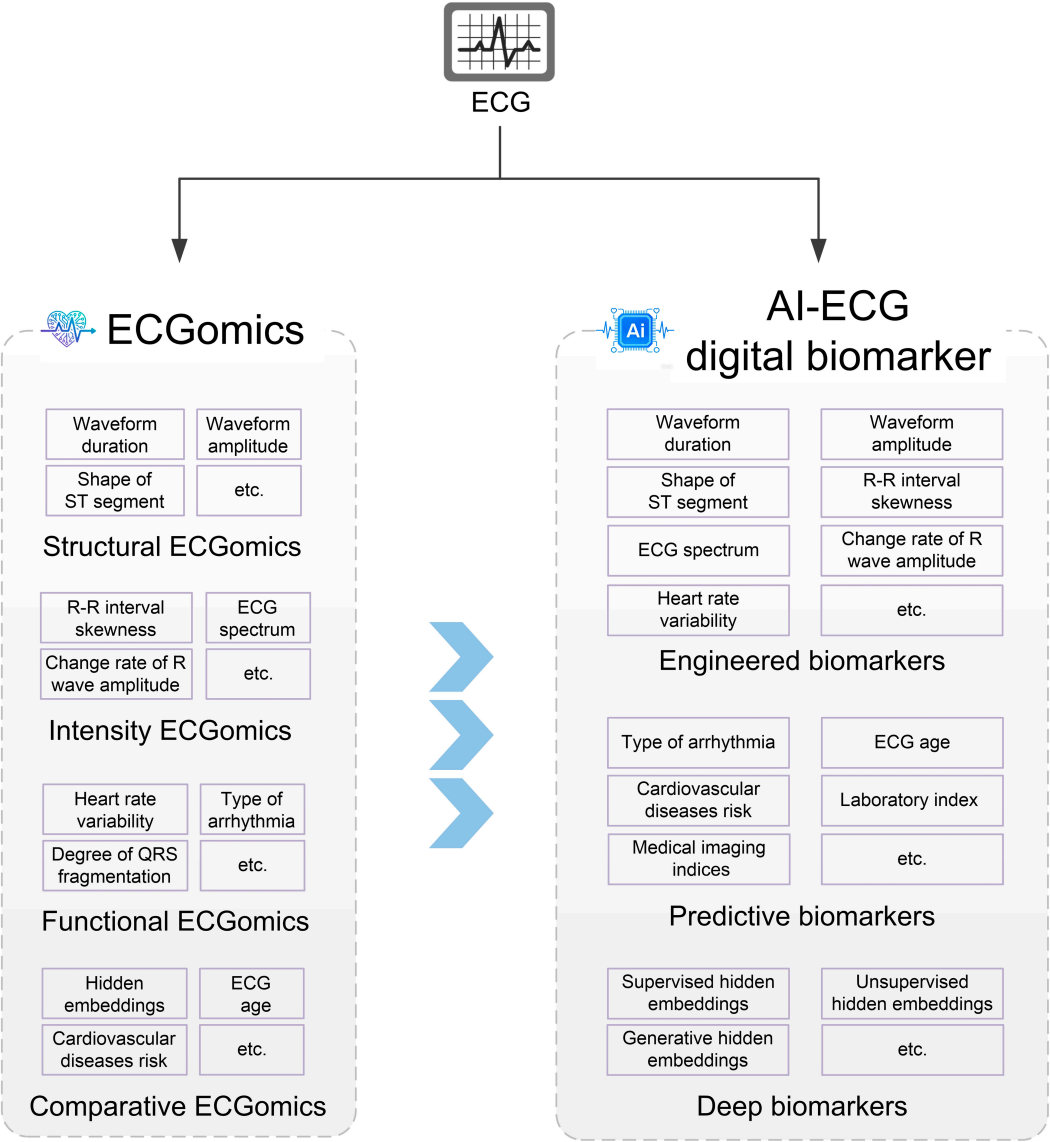
Artificial intelligence-electrocardiography (AI-ECG) digital biomarker overview. This illustrates the classification logic from the underlying raw features to the high-level predictive model, mainly including: engineered biomarkers (corresponding to structural, intensity, and functional dimensions, such as waveform duration, spectrum, and heart rate variability), predictive biomarkers (corresponding to comparison dimensions, such as cardiac age and disease risk prediction), and deep biomarkers based on deep learning (such as various hidden layer embedded features).

Figure 3 delineates a hierarchical taxonomy of AI-ECG digital biomarkers, categorizing them into 3 progressive layers that align with the ECGomics framework, comprising Structural, Intensity, Functional, and Comparative dimensions. This systematic deconstruction bridges traditional clinical interpretation with advanced computational phenotyping, providing a comprehensive understanding of disease.

At the foundational level, engineered biomarkers encompass the Structural, Intensity, and Functional dimensions of ECGomics. These are derived through expert-defined rules and include morphological parameters such as waveform duration and amplitude, which quantify the physical assembly of the cardiac cycle. Concurrently, these markers capture nonlinear dynamics and autonomic regulation through metrics like the ECG spectrum, R–R interval skewness, and heart rate variability, thereby providing a multidimensional assessment of cardiac conduction integrity and energy distribution.

The middle tier consists of predictive biomarkers, which primarily align with the Comparative ECGomics dimension. By leveraging AI-enabled association features, these biomarkers benchmark individual electrophysiological signals against vast population datasets. This comparative analysis facilitates high-level clinical inferences, including the determination of “ECG age”, the stratification of cardiovascular disease risk, and the cross-modal prediction of laboratory indices and medical imaging features that transcend traditional visual analysis.

Finally, the architecture culminates in deep biomarkers, representing the pinnacle of data-driven representation. These biomarkers consist of hidden embeddings—latent features extracted via supervised, unsupervised, and generative DL architectures. Unlike engineered features, deep biomarkers encapsulate complex, high-dimensional patterns within the cardiac signal that are often imperceptible to the human eye, offering a granular, digital representation of the heart’s underlying physiological state.

### Analytical workflow: From extraction to application

The conceptualization and practical execution of the ECGomics framework are operationalized through a rigorous 3-stage analytical pipeline consisting of multidimensional extraction, systematic biomedical mapping, and clinical translation applications.

In the initial extraction stage, we implement a sophisticated hybrid strategy that harmonizes clinical interpretability with high-dimensional data expression. This process utilizes our proprietary FeatureDB framework to extract a structured vector of expert-defined morphological and physiological metrics, such as traditional wave intervals and amplitudes, while simultaneously employing the ECGFounder foundation model to derive latent digital biomarkers. By processing raw signals through the Net1D architecture, we capture high-dimensional hidden-layer embeddings that encompass subtle, subclinical signal perturbations often invisible to human observers. This integration of a glass-box expert engine and a deep representation engine ensures that the resulting ECGomics serves as a comprehensive digital biomarker of cardiac and systemic health.

Building upon this data foundation, the biomedical analysis stage serves as a conceptual bridge that maps ECGomics features onto a broad landscape of clinical and biological information, closely paralleling the integrative logic of multiomics research. This phase involves deep phenotyping through statistical correlation and integrative modeling to link ECGomics vectors with diverse clinical layers, ranging from laboratory biomarkers, such as NT-proBNP, to cardiac imaging metrics, including left ventricular ejection fraction. Furthermore, this stage facilitates the identification of electrogenetic signatures by correlating deep representations with genomic data, thereby revealing the underlying genetic determinants of cardiac electrical patterns. Unlike traditional ECG, which focuses predominantly on localized cardiac pathology, the ECGomics paradigm expands into systemic mapping, positioning the heart as a holistic sensor capable of reflecting metabolic, renal, and endocrine dysfunctions. By training on the concatenated vector of expert and deep features, the model performs personalized risk stratification and disease detection.

The final application stage translates these systemic insights into actionable clinical decisions through robust predictive modeling and personalized medicine strategies. By utilizing advanced machine learning algorithms, notably XGBoost, the framework effectively handles the heterogeneous and high-dimensional nature of the fused feature sets to identify and forecast diverse clinical outcomes. This modeling paradigm enables precise and personalized risk stratification across a wide spectrum of conditions, ranging from acute cardiovascular events to noncardiovascular systemic diseases, such as chronic kidney disease. Ultimately, the ECGomics pipeline facilitates a transition toward proactive, precision care, supporting the identification of novel digital biomarkers for noncardiac diseases’ opportunistic screening (e.g., chronic kidney disease and anemia), the design of individualized behavioral interventions, and the high-resolution prediction of long-term disease trajectories for global patient management.

### Online tool development: ECGomics analysis platform

**Fig. 4.**
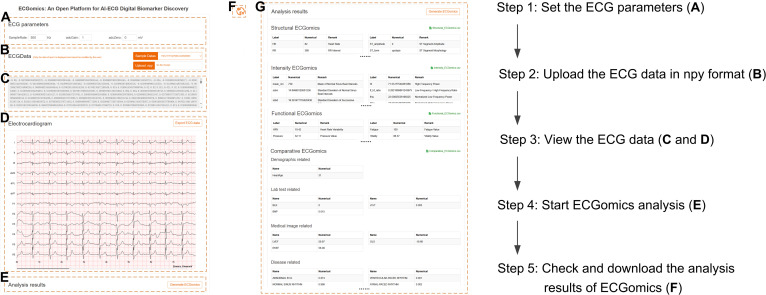
User interface and workflow of the ECGomics platform. (A) Signal acquisition configuration (SampleRate, adcGain, and adcZero). (B) Data selection via sample library or custom .npy upload. (C) Raw electrocardiography (ECG) numerical display. (D) Twelve-lead ECG visualization with export function. (E) “Generate ECGomics” execution control. (F) Language switching (Chinese/English). (G) Output panel presenting results across 4 ECGomics dimensions, with CSV export option.

**Fig. 5.**
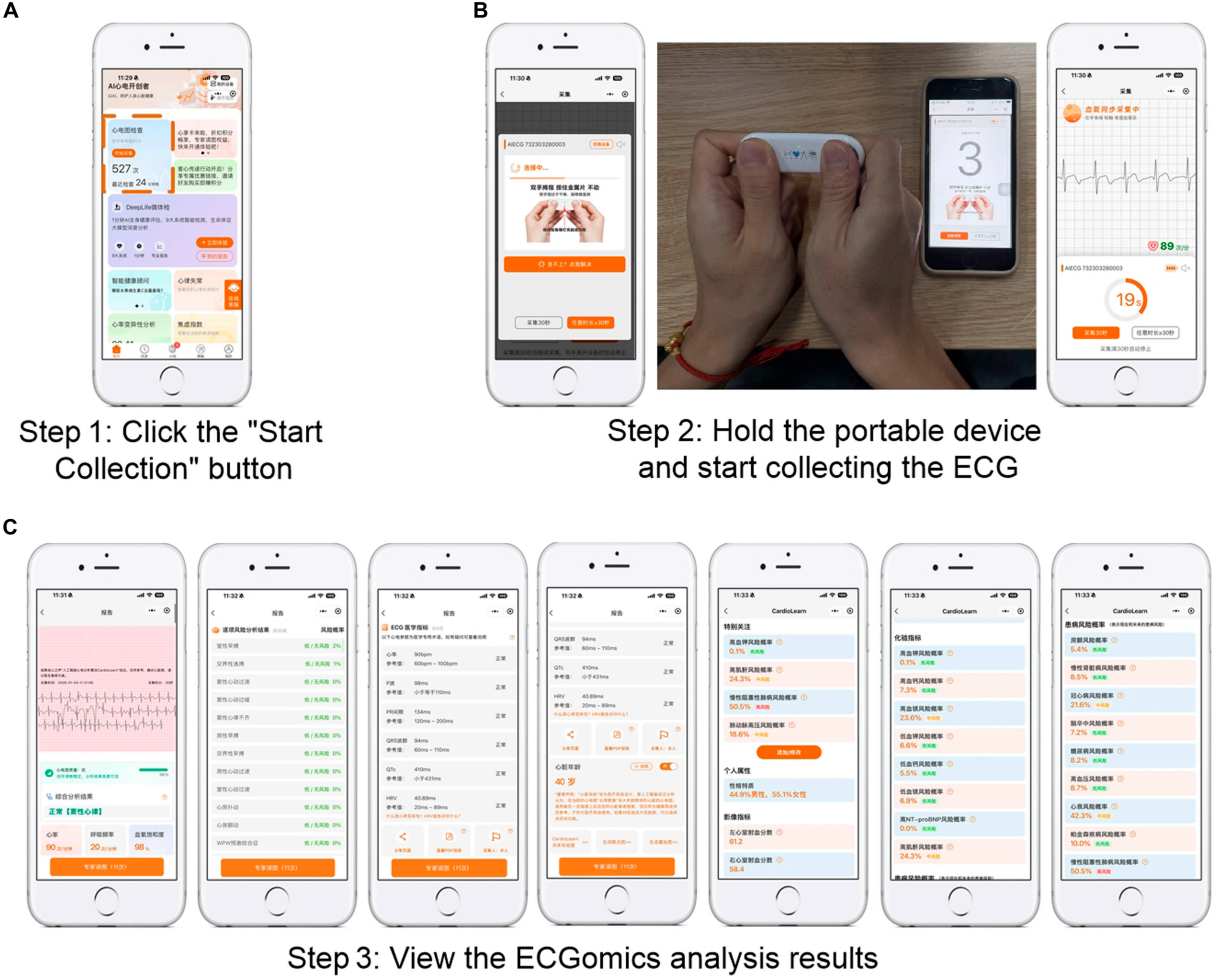
Workflow for portable electrocardiography (ECG) collection and ECGomics analysis. (A) Initiate data acquisition via the mobile application. (B) Record ECG signals using the handheld device with real-time waveform feedback. (C) Automated analysis generates a comprehensive report including waveform visualization, rhythm parameters (e.g., heart rate, PR interval, and QRS duration), and predictive metrics such as cardiac age and risk assessment.

To facilitate the broad adoption of the proposed framework and bridge the gap between theoretical research and clinical utility, we have developed a dedicated web-based (Fig. 4) and phone-based (Fig. 5) platform titled ECGomics Analysis, accessible at https://github.com/PKUDigitalHealth/ECGomics. This online tool serves as a practical, open-access gateway for the global research community, allowing for the high-throughput implementation of the ECGomics pipeline without requiring local high-performance computing clusters or DL expertise. The platform is engineered to automate the transition from raw physiological signals to structured digital phenotypes by integrating our core backend engines, including the Net1D-based FeatureDB protocol for expert-driven morphological characterization and the ECGFounder foundation model for latent representation mining. Through a user-friendly interface, researchers can upload raw ECG recordings and receive a comprehensive deconstruction of the signal across the structural, intensity, functional, and comparative dimensions.

A primary objective of this development is to ensure data transparency and foster collaborative innovation in the field of precision medicine. Upon completion of the automated analysis, the platform allows users to download the full suite of extracted ECGomics features as structured datasets. These downloadable resources include both clinically interpretable metrics and high-dimensional hidden-layer embeddings, enabling users to perform independent downstream investigations such as large-scale phenotype association studies or the discovery of novel electrogenetic biomarkers. By providing this standardized tool for feature extraction and digital biomarker identification, the platform democratizes access to advanced AI-enabled ECG analysis, supporting the identification of systemic health indicators and the high-resolution prediction of disease trajectories across diverse patient populations.

### Usage instructions

To facilitate the broad adoption of the proposed framework and bridge the gap between theoretical innovation and clinical utility, the ECGomics paradigm is operationalized through a web-based ecosystem (Fig. 4) and a phone-based ecosystem (Fig. 5), ECGomics Analysis, available at https://github.com/PKUDigitalHealth/ECGomics. This interactive platform serves as a practical implementation of the ECGomics pipeline, providing a high-throughput interface for the intuitive exploration and real-time visualization of multidimensional cardiac signatures. By lowering the technical barriers associated with complex signal processing and DL, the platform enables researchers and clinicians to upload raw ECG recordings—or use preloaded benchmark examples—to systematically deconstruct the cardiac signal into a comprehensive and structured feature space.

Furthermore, to address scenarios where standard ECG signals are difficult to obtain, we have developed a mobile ECGomics platform compatible with portable single-lead devices. This platform, integrated within the WeChat mini-program ecosystem, leverages portable sensors for signal acquisition and is capable of performing rapid, automated extraction and delivery of AI-ECG digital biomarkers in near real time.

#### Option 1: Use web-based ECGomics platform

The following steps define the end-to-end functional chain of the ECGomics platform, a specialized system for the systematic discovery and extraction of AI-ECG digital biomarkers.

##### Step 1: Configuration of the precision parameter

This foundational stage ensures that the platform accurately interprets the physical properties of the electrocardiographic signal. Users configure critical acquisition parameters, including the sampling rate (defining temporal resolution), ADC gain (normalizing signal amplitude), and zero-point voltage (establishing the baseline calibration). The alignment between these digital settings and the physical acquisition hardware is paramount to maintain signal fidelity during downstream analysis (Fig. 4A).

##### Step 2: High-fidelity data ingestion via .npy format

The platform utilizes the .npy format to facilitate data input, a choice that ensures the efficient storage of multidimensional numerical arrays while preserving the raw integrity of the ECG signal. In the data management module, users may either perform a localized upload of their proprietary files via the “upload npy file” interface or utilize standardized “Sample Data” for benchmark testing (Fig. 4B). Upon submission, the platform executes automated verification of file integrity to establish a robust dataset for the analytical engine.

##### Step 3: Multilead visualization and signal verification

To bridge the gap between raw data and clinical insight, the uploaded signal is rendered into an intuitive 12-lead ECG waveform visualization. This interface allows researchers to perform a preliminary qualitative review of rhythm patterns and morphological characteristics. Furthermore, an “Export ECG data” function is provided, enabling users to archive the digitized waveforms or conduct secondary independent validation (Fig. 4C and D).

##### Step 4: AI-driven execution of the ECGomics engine

The core of the discovery process is initiated by executing the “Generate ECGomics” command. This triggers the platform’s proprietary AI algorithms to perform a multidimensional deconstruction of the cardiac signal. The engine extracts digital biomarkers across 4 interconnected dimensions: Structural ECGomics, Intensity ECGomics, Functional ECGomics, and Comparative ECGomics (Fig. 4E).

##### Step 5: Dimensional result synthesis and research

The workflow culminates in the presentation of a structured ECGomics report, which categorizes biomarkers into modules such as demographics-related traits (e.g., heart age), laboratory test correlations, and disease probability scores. To support further scientific inquiry, each analytical module allows for data extraction in csv format. This ensures seamless compatibility with external statistical software, facilitating high-level research integration and large-scale clinical population studies (Fig. 4F).

#### Option 2: Use phone-based ECGomics platform with portable devices

##### Step 1: Procedural activation

Upon launching the mobile health application (WeChat mini program), the user accesses a centralized dashboard that integrates personal health metrics with historical ECG records. The diagnostic sequence is initiated by engaging the prominent “Start Collection” interface. This action triggers the system’s initialization phase, transitioning the interface into a preparatory mode that displays essential operational guidelines to ensure procedural compliance before hardware coupling (Fig. 5A).

##### Step 2: Synchronized signal acquisition

Following initialization, the mobile terminal automatically establishes a secure near-field communication link (typically via Bluetooth) with the portable ECG sensor. Once pairing is confirmed, the interface guides the user to maintain a standardized bimanual posture to ensure optimal electrode–skin conductivity. The device then captures ECG in real time, synchronizing the raw data stream with the mobile unit, while a dynamic visual indicator (e.g., collection countdown) provides immediate feedback on the progress of acquisition (Fig. 5B).

##### Step 3: Cloud-based ECGomics analysis and result delivery

Upon completion of signal acquisition, the raw data are securely transmitted to the CardioLearn cloud service platform for processing. The cloud infrastructure deploys professional AI algorithms to execute a multidimensional ECGomics analysis on the dataset. The processed results are rapidly returned to the mobile interface as a structured report, encompassing high-fidelity waveform visualization, quantitative rhythm parameters, and functional cardiac assessments, thereby providing the user with immediate, interpretable insights into their cardiac health (Fig. 5C).

#### Option 3: Access via API integration

In addition to the web-based and mobile deployment modes, ECGomics provides a RESTful application programming interface (API) interface for programmatic access. This allows integration into third-party research pipelines or clinical information systems.

Users submit ECG waveform data in JSON format via HTTP POST requests. The required parameters include:

• ecgData: raw ECG waveform data (JSON array format)
• ecgSampleRate: sampling frequency (Hz)

##### Step 1: Data preparation

The raw ECG waveform must be formatted as a structured JSON object prior to submission. The input payload requires 2 fields: ecgData, representing the ECG waveform sequence as a JSON array, and ecgSampleRate, specifying the sampling frequency in hertz. The waveform data should be digitized and stored in a text-based JSON format to ensure compatibility with the API specification.

##### Step 2: API invocation

Once the data are properly formatted, they are transmitted to the ECGomics server via an HTTP POST request with the content type set to application/json. The server end point processes the submitted waveform automatically, performs multidimensional ECGomics feature extraction, and generates predictive outputs. Upon successful execution, the API returns structured results in JSON format, including engineered biomarkers, deep representation features, and model-derived predictive metrics.

An example of the API invocation code is provided below. import requestsimport jsonif __name__ == ’__main__’:with open(’ECG.txt’, ’r’) as fr:ecg_data = json.load(fr) data = {’ecgData ’: ecg_data,’ecgSampleRate ’: 121}datas = json.dumps(data)

url = http://110.157.241.24:18012/ECGOmics
headers = {’Content-Type’: ’application/json’}response = requests.post(url=url, headers=headers, data=datas)response_data =json.loads(response.text)print(response_data)

## Results

The ECGomics framework has been rigorously applied to a series of representative scenarios, demonstrating its versatility and robustness as a multidimensional analytical paradigm for both cardiac-specific and systemic health assessment.

### Atrial fibrillation detection

In our previous work, we developed ENCASE [29], a 2-stage analytical method that epitomizes the integration of expert-driven morphological characterization and data-driven deep representations. By utilizing short, single-lead ECG recordings from the PhysioNet 2017 Challenge, this approach achieved a superior F1 score of 0.825 across diverse rhythm categories (Normal, AF, and Other). A critical finding of this study was through feature attribution analysis, which revealed that expert-defined morphological features and deep neural representations provide complementary physiological information. This synergy allowed the model to maintain high diagnostic accuracy even in noisy or brief signal segments, establishing the feasibility of the ECGomics framework for reliable, real-time arrhythmia screening.

### Predicting atrial fibrillation recurrence after cryoablation

To explore the prognostic potential of the framework, we investigated the prediction of atrial fibrillation recurrence following cryoablation therapy [30]. By analyzing standard 12-lead ECGs from a cohort of 201 patients, we constructed predictive models that integrated baseline AF subtypes with digital biomarker—the high-dimensional differences between pre- and postprocedural deep representations. The optimized XGBoost model achieved an area under the receiver operating characteristic curve of 0.872 and an overall accuracy of 0.902, substantial outperforming traditional clinical risk scores. These results underscore the unique ability of ECGomics to extract biomarkers from subtle shifts in the cardiac electrical activity, providing a quantitative basis for individualized risk assessment and the optimization of postablation follow-up strategies.

### Detection of severe coronary stenosis in apparently normal ECGs

The ECGomics framework has also demonstrated remarkable efficacy in uncovering hidden cardiovascular risks in apparently normal ECGs [31]. In a study involving 392 patients, we applied deep transfer learning to identify individuals with severe coronary stenosis who displayed no overt abnormalities under conventional clinical inspection. While standard ECG interpretation showed limited sensitivity (0.545), the integration of deep ECGomics features with multimodal clinical variables improved the sensitivity to 0.848 with an area under the receiver operating characteristic curve of 0.847. This application highlights the framework’s strength in Comparative ECGomics, where the model learns to detect subtle subclinical perturbations that escape human observation, effectively serving as a noninvasive screening tool for high-risk coronary artery disease.

### Maternal health management based on ECGomics

Extending the reach of ECGomics to specialized populations, we evaluated the reliability and validity of a novel AI-enabled portable device, WenXinWuYang, for cardiac monitoring in pregnant women [32]. Pregnancy introduces significant physiological changes that increase the cardiac load, yet continuous monitoring is often hindered by the inconvenience of frequent hospital-based 12-lead ECGs. In a comparative study of 99 pregnant women across all trimesters, the ECGomics-based system demonstrated high diagnostic consistency (>0.900) and strong correlation with clinical gold standards for heart rate (*r* = 0.957) and QT intervals (*r* = 0.774). The system achieved a sensitivity of 0.842 and a specificity of 0.975 in detecting arrhythmias, proving that the integration of DL-based representation with portable hardware can provide professional-grade monitoring in real-world maternal care. This application confirms that ECGomics can effectively facilitate the transition toward proactive, home-based health management for vulnerable populations.

### Summary of above studies

Collectively, these case studies validate the wide-ranging utility of the ECGomics paradigm across diagnostic, prognostic, and risk-stratification tasks. Three core strengths are consistently evidenced: First, the synergistic integration of morphological, intensity, and deep representations significantly enhances predictive power over traditional methods; second, the use of interpretability tools (e.g., shapley additive explanations) bridges the gap between AI-driven discovery and clinical trust; and third, the framework possesses a unique capacity to reveal hidden systemic and cardiovascular markers that are otherwise imperceptible. Together, these milestones reinforce ECGomics as a transformative, systematic paradigm for the future of precision cardiovascular medicine.

## Discussion

### Theoretical implications of ECGomics

ECGomics introduces a conceptual shift in the epistemological framing of electrocardiographic analysis. Traditionally, ECG interpretation has been anchored in visually guided clinical reasoning or task-specific feature extraction. While these paradigms have proven clinically valuable, they treat ECG primarily as a diagnostic waveform rather than as a structured biological data system. ECGomics redefines ECG signals as high-dimensional physiological representations that can be formalized, standardized, and systematically extended across analytical contexts. From a methodological perspective, the theoretical contribution of ECGomics lies in its formalization of ECG-derived information into a hierarchical representation taxonomy. By organizing structural, intensity-based, functional, and comparative dimensions under a unified analytical architecture, the framework shifts the focus from isolated parameter extraction to representation-level modeling. This transition aligns ECG research with the foundational principles of omics sciences, where biological complexity is captured through structured, multilayered profiling systems rather than through single-parameter diagnostics.

Importantly, ECGomics also reconciles interpretability and representation learning within a single framework. Conventional feature engineering prioritizes clinical interpretability but may overlook latent high-dimensional patterns, whereas DL models capture complex representations but often lack structural transparency. By embedding expert-defined electrophysiological descriptors alongside AI-derived latent embeddings, ECGomics establishes a modular system in which interpretable biomarkers and data-driven representations coexist. This integration provides a theoretical pathway for bridging hypothesis-driven cardiology and data-driven biomedical discovery. At a broader level, ECGomics reframes the electrocardiogram as a scalable digital phenotype. Through standardized representation and population-level benchmarking, ECG signals can be integrated with systemic health indicators, longitudinal monitoring systems, and multimodal biomedical data to enhance overall health. This reconceptualization supports the development of interoperable digital biomarker ecosystems and positions ECG as a central component of precision cardiovascular medicine.

### Methodological comparison with expert-driven and DL-driven workflows

**Fig. 6.**
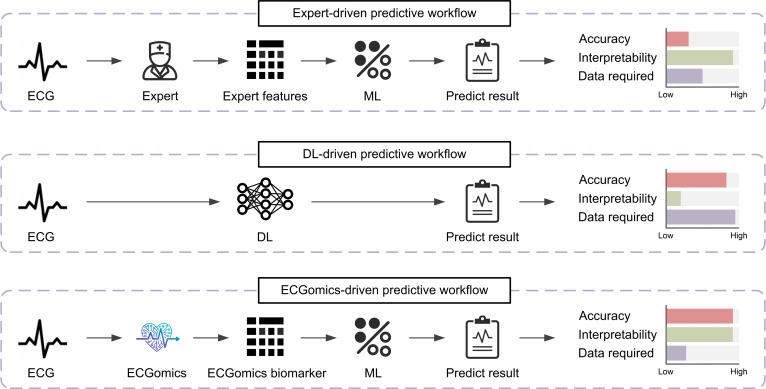
Comparison of different electrocardiography (ECG) prediction workflows. This figure illustrates the methodological evolution and performance trade-offs across 3 ECG analysis paradigms. (Top) The Expert feature-driven workflow utilizes handcrafted features based on clinical knowledge, offering high interpretability but limited accuracy due to the omission of latent signal patterns. (Middle) The deep learning (DL)-driven workflow achieves superior accuracy through end-to-end feature learning but functions as a “black box” with low interpretability and high data dependency. (Bottom) The proposed ECGomics-driven workflow synergizes expert-defined morphology with deep latent representations. As shown in the bar charts (right), the ECGomics approach achieves a high balance between accuracy and interpretability while maintaining a low data requirement, demonstrating its superior utility for precision medicine applications.

As illustrated in Fig. 6, we compared 3 distinct ECG predictive workflows to highlight the unique advantages of the ECGomics-driven approach in balancing accuracy, interpretability, and data required.

Expert-driven workflow: This traditional approach relies on manually defined morphological features extracted by clinical experts. While it offers the highest level of interpretability due to its grounding in established physiological rules, it suffers from limited accuracy as it fails to capture the high-dimensional latent information within the signal. However, it remains highly efficient in terms of data requirement, necessitating only a small, well-annotated dataset for model training.

DL-driven workflow: In contrast, the end-to-end DL approach utilizes raw ECG signals to achieve superior accuracy by automatically learning complex patterns. However, this gain in performance comes at the cost of being a “black box”, leading to very low interpretability. Furthermore, it is highly data-intensive, requiring massive amounts of labeled data to reach its full potential.

ECGomics-driven workflow: The proposed ECGomics paradigm serves as a synergistic middle ground. By integrating the glass-box interpretability of expert features with the powerful expressive capacity of deep latent representations (via models like ECGFounder), it achieves an accuracy comparable to DL-driven methods while significantly outperforming them in interpretability. Crucially, by leveraging pretrained foundation models, the ECGomics workflow maintains a relatively low data requirement for downstream tasks, making it a highly practical and robust solution for personalized medicine.

The high-dimensional deep embeddings generated during the ECGomics data extraction phase differ fundamentally from traditional expert-defined features. While engineered features (e.g., PR interval, QRS duration, and spectral metrics) are designed based on established physiological knowledge and predefined rules, deep embeddings are learned representations derived from large-scale self-supervised training. These embeddings capture latent, nonlinear, and higher-order dependencies within ECG signals that may not be explicitly encoded by expert features. Conceptually, deep embeddings function as a learned coordinate system in which ECG signals are mapped into a continuous representation space, enabling more flexible decision boundaries for downstream machine learning algorithms.

### Clinical implications and real-world deployment

ECGomics has significant clinical implications by extending the analytical value of ECG signals beyond traditional interpretation toward scalable, data-driven cardiovascular phenotyping and risk stratification. A growing body of evidence demonstrates that AI-enabled ECG analysis can meaningfully impact clinical decision-making and population health management [33]. For instance, AI-based ECG models achieve moderate to good discriminatory performance in predicting heart failure, suggesting potential utility as a noninvasive and cost-effective screening tool that could be deployed at point of care or in resource-limited settings [34].

Clinically oriented AI-ECG applications have also been developed for structural and functional cardiac assessment. Real-world studies have demonstrated that AI models trained on large ECG cohorts can detect asymptomatic structural heart disease with considerable accuracy, outperforming expert clinicians and flagging patients who may benefit from targeted diagnostic follow-up such as echocardiography [35]. Beyond specific disease detection, AI-augmented ECG methods have been validated in prospective cohort settings for outcome prediction in acute cardiovascular conditions. For example, DL algorithms applied to ECG recordings have been shown to independently predict adverse outcomes including in-hospital mortality among patients with acute heart failure, even after adjustment for established clinical scores and imaging biomarkers [36].

The real-world deployment of ECGomics also aligns with the increasing integration of wearable and consumer health technologies into clinical workflows [37]. Large-scale AI models trained on extensive ECG repositories have been adapted to interpret single-lead wearable device ECGs, illustrating feasibility for community-based screening and longitudinal monitoring outside traditional clinical environments [38].

Collectively, these developments underscore that multidimensional ECG representation frameworks, like ECGomics, can support a range of clinical and public health applications: from early disease detection and risk stratification to continuous monitoring and personalized prognostication. Future implementation research focused on workflow integration, clinical validation in diverse populations, and regulatory adherence will be key to realizing ECGomics’ potential in standard care.

### Limitations and future directions

Several limitations should be acknowledged. First, although the ECGomics taxonomy provides a structured representation framework, the current implementation may not exhaustively capture all possible electrophysiological dimensions. Future work could refine and expand the taxonomy to incorporate additional signal modalities or mechanistic layers. Second, while the framework integrates expert-defined descriptors and AI-derived representations, the interpretability of deep latent embeddings remains an ongoing challenge. Further methodological development is required to enhance transparency, attribution, and physiological grounding of representation learning components. Finally, while ECGomics establishes a theoretical and computational foundation, its long-term clinical impact will depend on rigorous prospective trials, workflow integration studies, and health-economic evaluations. Continued interdisciplinary collaboration will be essential to translate the framework into sustained improvements in cardiovascular outcomes.

## Conclusion

This work establishes ECGomics as a systematic paradigm that integrates morphological, associative, and deep representations to bridge the gap between traditional feature engineering and end-to-end DL. Validated through diverse case studies—ranging from arrhythmia detection to identifying occult coronary stenosis—ECGomics demonstrates superior diagnostic accuracy and interpretability while maintaining data efficiency. These capabilities allow the framework to uncover subtle physiological patterns essential for personalized medicine. Despite these strengths, challenges regarding data scale, annotation quality, and the need for rigorous multicenter validation persist. Future advancements must focus on refining model interpretability and integrating ECGomics with multiomics (e.g., genomics) and multimodal clinical data. Collectively, these developments position ECGomics as a foundational paradigm to advance precision cardiovascular diagnosis, risk stratification, and therapeutic decision-making.

## Data Availability

The ECGomics analytical platform and related implementation resources are publicly available at: https://github.com/PKUDigitalHealth/ECGomics.
